# Pathology of Fevers

**Published:** 1855-10

**Authors:** J. G. Westmoreland

**Affiliations:** Atlanta, Georgia


ATLANTA

Medical and Surgical Journal.

VOL. I.] OCTOBER, 1855. [NO. 2.

### ORIGINAL COMMUNICATIONS.

ARTICLE I.—*Pathology of Fevers.* By J. G. WESTMORELAND, M. D., of Atlanta, Georgia.

The period of duration in Idiopathic fevers, generally determined by the diagnostic distinctions, is of immense importance in the study of these very common and sometimes dangerous affections.

Almost every disease has its specific time of duration, and it is only necessary to ascertain the nature of the disease in order, under ordinary circumstances, to determine the length of time required for the arrest or termination of the malady.

The various forms of fever observe a uniform period of duration, according to the character found to exist in each particular variety. Not only so, but in the several varieties, all cases of the same kind will be found to resemble each other in exacerbations, remissions, stages, &c. The different varieties, however, differ greatly from each other in these particulars. The uniformity of symptoms thus observed in all the cases of the same kind of fever must be governed by the same pathological condition. It is equally evident that the pathological derangement of the several varieties must differ in each, either in the part affected or in the nature of that affection.

We have for the varieties, as designated by some of the general characteristics, Intermittent, Remittent, and Continued fevers.

Then we have the continued form divided by less distinct peculiarities into what may, for want of a more appropriate name, be termed Inflammatory Remittent, of two weeks, and Typhoid fever of three weeks' duration.

These distinctive characters do not depend upon mere accidents, occuring so as to vary the symptoms of the same disease. No. The particular caste or type is given by the radical lesion or impression; and although the derangement may be of the same character in Remittent as in Continued fever, yet the seat of the disturbance may not be in the same part of the organism.

The complicated structure of the great nervous centers, together with their diversified functions, renders it possible that very dissimilar trains of symptoms may result from precisely the same character of disease, located, too, only a few inches from each other. It is known that the action of the optic nerve, for instance, may be paralized, while the other functions connected with the brain may remain perfect. So with other portions of the nervous system. When disease occurs in any portion of the cerebro-spinal axis, those functions alone are disturbed which are governed directly or indirectly by that particular portion; and the nature of that derangement depends upon the character of the impression made upon the nervous center. If an undue excitement is communicated by the morbific agent, the functions of that portion of the nervous system will be exalted and irregular; if of a nature to depress the nervous energy, a corresponding modification of the functions may be expected.

As regards the accompanying symptoms of Typhoid fever, such as ulceration, &c., of the mucus membrane and follicles of the intestinal canal, they must be looked upon as secondary or incidental disturbances. That these tendencies exist in the bowels is not sufficient reason for ascribing to them the controlling influence in this fever, notwithstanding they may be found in a majority of cases; for, while there are other local lesions which, though they may not be so frequently found, are nevertheless equally characteristic of the disease. Phlegmonous inflammation of the parotid glands, and certain eruptive diseases of the skin, are among those which occasionally occur. It is presumed that no one will say that petechiæ, sudamina, or parotidœa, are governing symptoms, or that they are necessary to the existence of the fever. These, together with bronchitis, and the lesions of the bowels before mentioned, that are found to exist, are the results, and not the causes, of the general derangement.

All diseases have a train of symptoms by which they are generally known, and yet the symptoms may be produced indirectly by the morbific agent acting on an organ or tissue not very intimately connected with that from which the symptoms arise. All poisons, whether solid, liquid, or gaseous, have a specific tendency to affect certain organs, and by whatever channel they may be introduced into the system, they seek that upon which their influence is exerted. Hence we may anticipate, with some certainty, the action of certain poisons whose effects are known. To do so, however, it is important that we do not mistake the symptoms of secondary derangement for those of the primary disturbance.

Some poisons affecting the nervous system produce pain, restlessness, spasms, &c.; others, paralysis, coldness of the extremities and general surface, &c., &c.

Now, as a consequence of these nervous disturbances, various glandular and other derangements occur secondarily. So with the concomitants of fever; for even in Intermittent or Remittent fevers hepatic and gastric disturbances are common occurrences.

In these diseases no one will now perhaps contend that the organs, in which such incidental difficulties exist, are the subjects of radical disturbance. It is admitted, at least by a respectable number of the best modern pathologists, that the influence of the poison which induces these diseases is first felt by the spinal marrow.

The writer of this article entertains similar views in regard to the pathology of Typhoid fever; that is to say, a certain point in the nervous centers receives the first impression made on the system by the infectious cause of this fever. It however differs materially from the first mentioned fevers in point of duration, tendencies, &c., &c.; which fact is readily accounted for in the consideration of the difference in the points of invasion; and to the physical symptoms, observable only by careful examination of the whole spine, must we look to form correct diagnosis in these affections. Tenderness, on pressure, in the region of the first cervical vertebra is almost always felt by the subject of Typhoid fever, and in portions of the spine much lower, in the less malignant forms of fever, such as Miasmatic Remittent and Intermittent.

